# Hunting down NLRP3 inflammasome: An executioner of radiation-induced injury

**DOI:** 10.3389/fimmu.2022.967989

**Published:** 2022-10-24

**Authors:** Han Cheng, Lingling Chen, Minchun Huang, Jin Hou, Zhifeng Chen, Xiaojun Yang

**Affiliations:** ^1^ First School of Clinical Medicine, Southern Medical University, Guangzhou, China; ^2^ Department of Stomatology, Nanfang Hospital, Southern Medical University, Guangzhou, China

**Keywords:** NLRP3 inflammasome, inflammasome activation, radiation injury, therapeutic target, ROS, pyroptosis

## Abstract

Radiotherapy is one of the mainstream treatment modalities for several malignancies. However, radiation-induced injury to surrounding normal tissues limits its efficacy. The NLRP3 inflammasome is an essential mechanism of innate immunity that reacts to challenges from endogenous danger signals and pathological microbes. A growing body of evidence has demonstrated a key role of NLRP3 inflammasome in the pathogenesis of radiation-induced tissue injury. Despite accumulating evidence, the potential value of the NLRP3 inflammasome in the management of radiation-induced tissue injury is not adequately recognized. We conducted a literature review to characterize the relationship between NLRP3 inflammasome and radiation injury. By analyzing recent evidence, we identify NLRP3 inflammasome as one of the executioners of radiation-induced injury, since it responds to the challenges of radiation, induces cell pyroptosis and tissue dysfunction, and initiates non-resolving inflammation and fibrosis. Based on these concepts, we propose early intervention/prevention strategies targeting NLRP3 inflammasome in a radiation context, which may help resolve imperative clinical problems.

## Introduction

Radiotherapy is an effective treatment modality for various types of tumors. However, radiation-induced injury to normal tissues is an unavoidable adverse effect of radiotherapy. Despite ongoing advances in radiotherapy techniques that enable precise targeting of lesions, tissues adjacent to the irradiated field are liable to be affected by ionizing radiation. Typical examples of the adverse effects of radiotherapy include occurrence of carotid stenosis following head and neck radiotherapy, cardiovascular injury after thoracic radiotherapy, and gastrointestinal injury after pelvic and abdominal irradiation (1–6). In addition, indirect damage caused by the out-off field effects effect (capability of inducing similar responses in non-irradiated tissues) of radiation cannot be overlooked. For example, patients receiving radiotherapy to the head and neck region were reported to have a higher incidence of diarrhea (7). Similarly, irradiation of rat tongue was found to induce small intestine injury (8). These adverse effects may necessitate reduction in RT doses, limiting tumor control (9). Therefore, in-depth characterization of the mechanism of radiation injury and exploration of more effective management strategies are key imperatives.

The pathophysiological processes of different stages of radiation injury are well recognized. Initially, radiation causes DNA damage in cells, resulting in double-strand DNA breaks (DSBs) (10). Radiation-induced damage to DNA may occur directly *via* interacting with DNA molecules and causing DSBs, or indirectly by generating free radicals, such as reactive oxygen and nitrogen species (ROS, RNS), to cause base modification, and eventually leading to DSBs (11). The occurrence of DSBs induces the DNA-damage response (DDR), which initiates DNA repair by activating the MRN (Mre11-Rad50-Nbs1)-ATM (ataxia telangiectasia mutated)-H2AX (histone variant 2AX) signaling (11, 12). The transducer protein ATM, as well as ROS and other inflammatory stimuli, are capable of activating nuclear factor kappa-B (NF-κB) in a variety of manners, in order to better prepare cells for the stress (10, 13). DDR can also induce apoptosis or senescence if the damage cannot be repaired, meanwhile heightening immune surveillance for later scavenging of the remains (11). Moreover, other forms of cell death may still occur, irreparably damaged cells acutely generate robust amounts of pro-inflammatory factors, attracting and activating immune cells to the irradiated area. These accumulated inflammatory cells may potentiate tissue injury, establishing non-resolving inflammation and aberrant tissue remodeling, resulting in tissue injury (14–17). The intricate molecular mechanisms of radiation injury are yet to be fully elucidated. However, recent evidence suggests that ionizing radiation induces activation of inflammasome which functions as the “executioner” for radiation, mediating certain kinds of tissue injuries.

Inflammasomes are macromolecular complexes that react to challenges such as exogenous or endogenous danger signals and pathological microbes, and mediate the maturation and release of interleukin-1β (IL-1β) and interleukin-18 (IL-18) as well as induction of pyroptotic cell death. The activation patterns and downstream responses of inflammasome make it an essential part of innate immunity, acting against various types of infections and injuries. Beyond host defensive response, recent studies pointed out the participation of several types of inflammasomes, including nucleotide-binding oligomerization domain (NOD)-like receptors, pyrin domain-containing protein 3 (NLRP3) and absent in melanoma 2 (AIM2) inflammasomes, in the pathology of radiation-induced normal tissue injury (18).

Among all inflammasomes so far identified, the nature of NLRP3 inflammasome is the most well characterized, which can be activated through several different mechanisms, namely the canonical, non-canonical and alternative activation (19). The canonical activation of NLRP3 inflammasome requires a two-step process, including a priming signal and an activation signal. Priming involves various pathogen-associated molecular patterns (PAMPs) (like bacterial LPS), damage-associated molecular patterns (DAMPs) (like eATP, uric acid, mtDNA, etc) and their recognition by TLRs, or through cytokines (like IL-1 and TNF-α) and their signalings (20). Priming leads to the activation of NF-κB, thereby increasing the transcription of inflammasome components (like NLRP3) and pro-IL-1β (18, 21, 22). The activation signals are most commonly induced by NLRP3 agonists, such as K^+^ efflux, Ca^2+^ signals, ROS, mitochondrial dysfunction and its released contents, and lysosomal rupture (also the released cathepsins). Upon activation, NLRP3 oligomerizes into a macromolecular inflammasome complex *via* recruiting adaptor protein apoptosis-associated speck-like protein containing a CARD (ASC) and effector molecule pro-caspase (cysteinyl aspartate specific proteinase)-1 (23–25). Activated inflammasome mediates the maturation of IL-1β and IL-18, as well as induces pyroptosis by cleaving its effector gasdermin-D (GSDMD) (26, 27). The non-canonical NLRP3 inflammasome activation entails human caspase-4/5 and murine caspase-11. Once lipopolysaccharide (LPS) from Gram-negative bacteria enters the cytoplasm, the aforementioned inflammatory caspases will undergo autoproteolysis and further activate GSDMD to form membrane pores, thereby inducing K^+^ efflux and triggering NLRP3 (19, 28). The alternative NLRP3 inflammasome activation shares no similarity with its canonical or non-canonical counterparts, which follows a complex TLR4–TRIF–RIPK1–FADD–CASP8 signaling (29).

The connections between radiation injury and inflammasome are particularly intriguing since regulation of inflammasome has been extensively studied and applied, suggesting a possibility to manipulate the complex in the context of radiation injury. For example, early intervention against radiation-induced inflammasome may alleviate tissue injury, deterring the establishment of chronic inflammation (30).

In the quest for more effective management strategies of radiation injury, this review seeks to make sense of the underlying mechanism of radiation-induced inflammasome activation and to explore the characteristics of such significant pathology.

## Current understanding of radiation-induced NLRP3 inflammasome activation

As previously described, ionizing radiation induces DSBs, DNA damage response, and oxidative stress. DSBs and DDR upregulate multi-functional signaling pathways such as NF-κB, leading to intensified immune response. Free radicals such as ROS and RNS are the major effectors of radiation damage which mediate the oxidation of biomolecules such as DNA, protein, lipids, and the regulation of several signaling pathways. With aggravation of tissue injury, some stressed and dying cells release contents that denote tissue injury to initiate damage-control-responses (10). In the development of these processes, a variety of signals are detected by the sensor protein NLRP3, leading to its activation.

### Radiation-induced oxidative stress leads to NLRP3 inflammasome activation

It has long been accepted that ionizing radiation causes tissue injury by disrupting the balance of reduction/oxidation system, characterized by over-production of free radicals and the induction of oxidative stress. ROS and RNS are the main types of free radicals produced on exposure to ionizing radiation, which are normally counteracted by the antioxidant system (10).

Upon irradiation, ROS is immediately produced from water radiolysis (31, 32). Moreover, ROS also possesses a self-amplifying cycle. By perturbing the mitochondrial electron transfer chain (ETC), ROS along with other free radicals can further inflict mitochondrial dysfunction, leading to enhanced generation of ROS (10). More importantly, ROS is widely accepted as a potent stimulant for NLRP3 inflammasome, which also serves significant roles in radiation biology. Radiation-induced reductive/oxidative enzymes (such as NOX, COX-2, NOS, and LOXs) produce ROS in a continuous manner, which repeatedly perturbs mitochondria and persistently provides stimulants, thereby maintaining NLRP3 inflammasome activation long after exposure to ionizing radiation (23, 31–35).

According to a study investigating the links between oxidative stress and NLRP3 inflammasome, increased ROS concentration results in the dissociation of thioredoxin-interacting protein (TXNIP) from oxidized thioredoxin-1 (Trx-1), followed by interaction of TXNIP with NLRP3, resulting in activation of the latter (36, 37). Moreover, as mentioned above, impaired mitochondria subsequently release more ROS into the cytosol, binding to NLRP3 and promoting its activation (38, 39).

It has been demonstrated that radiation directly activates the NLRP3 inflammasome in human monocyte-like cells (THP-1 cells) in a ROS-dependent manner. Eliminating ROS with N-acetylcysteine (NAC) was found to ameliorate NLRP3 activation and the release of IL-1β and IL-18 *in vitro* (40). Moreover, in *in vivo* conditions, radiation-induced NLRP3 inflammasome activation is accompanied by tissue oxidative stress, characterized by increased ROS levels, either generated by radiation or from perturbed mitochondria, leading to a hyper-oxidative state. Clearing free radicals with antioxidants, or through inducing antioxidant signaling (like Nrf2 signaling), can ameliorate inflammasome activation and alleviate animal radiation response (8, 41–43). Research above reveals that ROS-induced activation of NLRP3 inflammasome plays a critical role in the initiation and development of radiation-induced injury, thus NLRP3 may be a therapeutic target for radiation-induced injury.

### Radiation injury-derived DAMPs activate the NLRP3 inflammasome

In addition to ROS, other DAMPs are also generated in irradiated tissues. Ionizing radiation is known to inflict tissue injury and induce cell death, which is more constantly observed in actively dividing cells such as hematopoietic and epithelial cells (44). Lytic cell death leads to the release of cell contents, some of which serve as DAMPs and mediate the initiation of inflammation (17).

In recent studies, uric acid was shown to serve as a mediator of radiation-induced NLRP3 inflammasome activation in immune cells, mainly through endocytosis and damaging the lysosome membrane, resulting in the release of cathepsin B into cytosol, then triggering NLRP3 inflammasome (24, 44). Other DAMPs, such as extracellular ATP and mitochondrial DNA (mtDNA), may also contribute to this pathology. ATP released from damaged cells binds to the P2X7 receptor on immune cells, thereby inducing pannexin-1-dependent K^+^ efflux as well as the influx of extracellular DAMPs into the cytosol, leading to activation of NLRP3 inflammasome (23, 24, 39). Contents released from damaged mitochondria also may possess NLRP3 activating property, especially oxidized mtDNA, which exhibits a powerful stimulatory effect (23, 37, 45). These findings suggest that IR-induced damage may activate inflammasomes *via* a variety of mechanisms.

**Figure 1:**
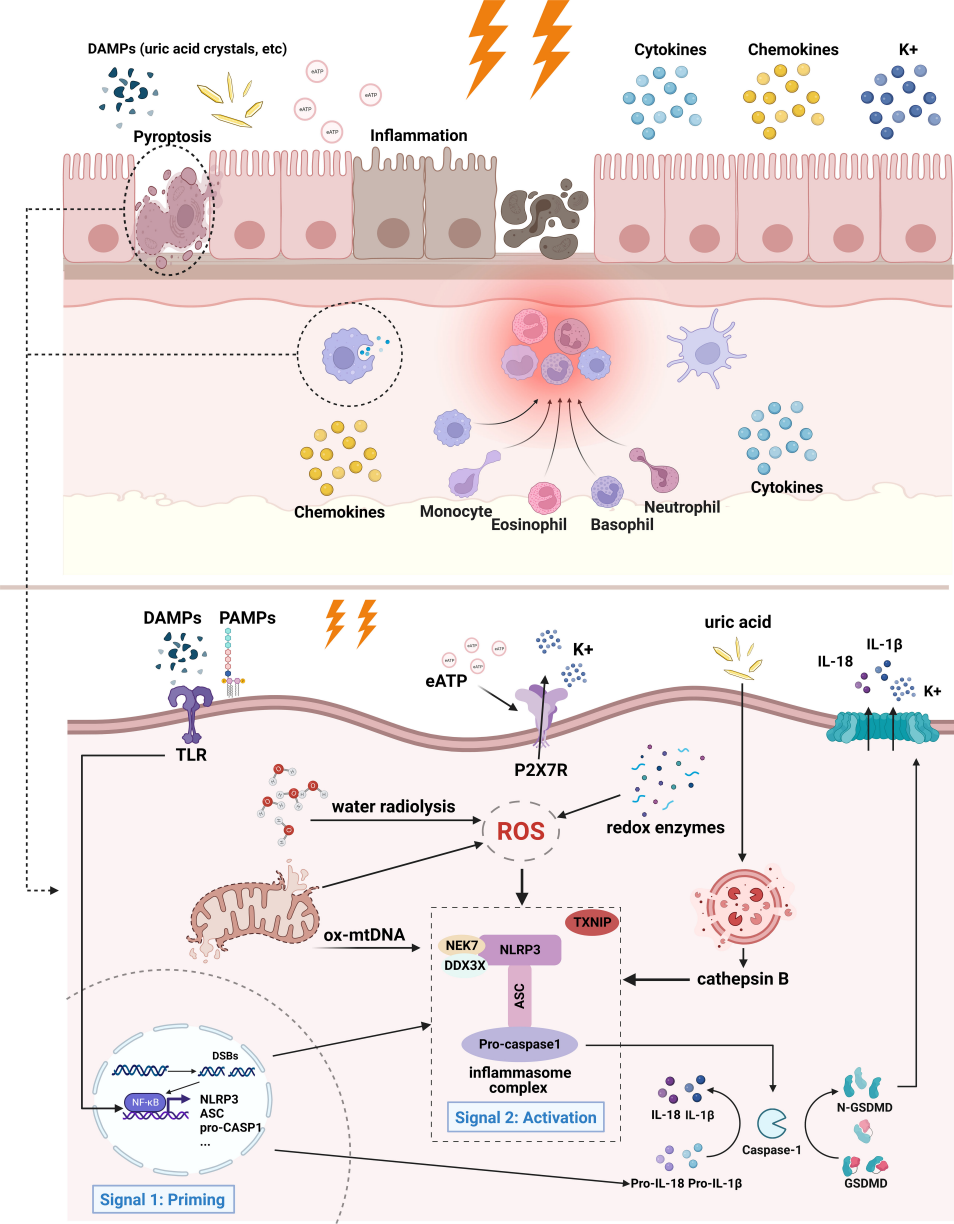
Mechanism of radiation-induced NLRP3 inflammasome activation. In the case of intestinal epithelial cells and infiltrating macrophages, irradiated cells initiate inflammatory responses and eventually undergo cell death. Adjacent cells may also be affected by inflammation. Specifically, extracellular PAMPs and DAMPs act as priming signals, binding to and inducing TLR signaling, thereby promoting the activation of NF-κB and the transcription of inflammasome components and pro-IL-1β. The activation signals entail ROS, K^+^ efflux, release of mitochondrial DNA, lysosomal rupture and cathepsin release, etc. ROS is produced from the radiolysis of water, redox enzymes, and dysfunctional mitochondria. Lysosomal membrane may be destabilized by particulate or crystalline structures (like uric acid crystal) and then lead to lysosomal rupture, releasing cathepsin B. Mitochondrial dysfunction gives rise to the release of mtDNA and cardiolipin. These activation signals stimulate NLRP3 inflammasome and lead to its oligomerization. Activated NLRP3 inflammasome mediates the maturation of IL-1β and IL-18, as well as induces pyroptosis by cleaving GSDMD. The downstream effects of NLRP3 inflammasome activation are characterized by the production of cytokines, chemokines, and recruitment of immune cells, followed by cell death. Of notes, the contribution of mtDNA and extracellular ATP to radiation-induced inflammasome has not yet been confirmed, therefore they are illustrated with fading arrows.

The main effectors of this process are immune cells, such as macrophages, dendritic cells, NK cells, T cells, and B cells, which lead to the secretion of pro-inflammatory factors, cell death, and release of cell contents (44, 46). Unsuccessful removal of these byproducts triggers the inflammatory cascade leading to tissue injury and chronic inflammation. A schematic illustration of the current understanding and potential mechanisms (eATP and mtDNA) of radiation-induced NLRP3 inflammasome activation is presented in **Figure 1**.

It is worth mentioning that the exact mechanisms of radiation-induced inflammasome activation may vary in different tissues and cells, thus may lead to divergent conclusions. Hence, we sorted and summarized the studies that investigated this process to better illustrate the differences in the physiological processes (**Tables 1**, **2**).

**Table 1:** *In vivo* studies involving radiation-induced NLRP3 inflammasome activation.

Animal	Radiation dose	Irradiated tissue	Observation period	NLRP3 activation-wise results	Possible connections	Reference
Male Wistar rats	7.5 Gy/day for five consecutive days	mouth	14 days	1. Increase in the NLRP3 and ASC protein levels and mRNA expression in irradiated tongues 2. Expression of pro-caspase-1 mRNA increased with irradiation followed by decreased protein levels 3. Increase in NF-κB mRNA and protein levels in the cytosol and nuclei of irradiated rat tongues	1. Increased mitochondrial LPO levels, mRNA, and protein levels of GPx, GSSG/GSH ratio, decreased GRd expression 2. Increased iNOS and i-mtNOS expression 3. Reduction in the expression of respiratory complexes I, III, and IV and the ATPase as well as a reduction in mitochondrial mass 4. Increase in autophagy/mitophagy markers Atg12, Beclin-1, and Nix in irradiated tongues	(41)
C57BL/6 mice (6 to 8 weeks old)	2, 4, 16 Gy	Lung	1, 4, 8 weeks	1. NLRP3 inflammasome was activated in mouse lungs by irradiation starting from 2 Gy, the extent of expression was not correlated with radiation dose 2. NLRP3 activation was continued for 8 weeks until sacrifice	1. NLRP3 activation was mainly found in the airway, rather than in the lung parenchyma	(40)
Human biopsy		Irradiated human artery	156 weeks (median) post-radiation therapy	1. Apoptosis and NLR signaling pathways are the most differentially expressed in irradiated human arteries 2. Marked elevation of genes encoding IL-1α and IL-1β, caspase-1 and NLRP3	1. Irradiated arterial biopsies had a marked increase in pro-caspase-1 and caspase-1	(30)
male Wistar rats (3-month-old)	7.5 Gy/day for five consecutive days	mouth	14 days	1. Increased protein level of NF-κB subunit p65 in the nucleus and cytosol, with increased expression of IL-1β, TNFα, and COX-2 2. Increased NLRP3 protein level in intestine, followed by decreased pro-caspase-1 and increased IL-1β	1. Increased ROS and NO levels in intestine tissue 2. Intestine show reduced expressions of respiratory complexes I, II, III and ATP synthase 3. decreased activity of antioxidant: GPx, GRd, Mn-SOD, with increased GSSG/GSH ratio	(8)
Adult female BALB/c mice	10 Gy	abdomen	6-15 days	1. On day 6, NLRP3, caspase-1, IL-1β and IL-18 mRNA levels were elevated in Intestinal cell, accompanied by increased caspase1 activity 2. IL-1, IL-8, MCP-1, TNF-α mRNA levels were elevated	1. Increased apoptosis and DNA damage (measured by γ-H2AX expression) were detected 2. IR increases SOD activity and concentration of GPx, GR and increases GSSG/GSH ratio in Intestinal cells on day 6	(47)
WT C57BL/6J male mice	9.5 Gy	Whole-body radiation	3 hours and 30 days	1. Cleaved-caspase-1 (p10) and IL-1β protein levels were induced in spleen cells 3 hours after irradiation, with a mild increase in NLRP3 protein level 2. Staining for cleaved-caspase-1 in spleen marginal zone cells were elevated 3 hours after radiation 3. Nlrp3 knockout was associated with significantly improved survival at 30 days after irradiation	1. Cleaved-caspase-1 were hardly observed in the white pulp cells of the spleen (rich in lymphocytes)	(48)
Male 5–7-week-old CD-1 mice, Male (caspase1 -/-) mice, Male 7-week-old C57BL/6J mice	0.5, 1, 2, 4 Gy	Whole-body radiation	1, 2, 4, 6 hours or 1, 3, 7, and 14 days	1. A dose-dependent increase in cleaved-caspase-1(p10) levels were examined in spleen cells 1 day after radiation but was not detectable at 1 or 4 hours after radiation 2. 2 Gy radiation induced increases of cleaved-caspase-1 sustained for 7 days and returned to baseline levels on day 14 3. PI-Annexin V double positive spleen cells were increased 4 hours after 2 Gy radiation, reached highest level on day 1 and returned to baseline on day 14 4. Caspase-1 deficient mice show increased surviving spleen cells 1 day after 2 Gy radiation, as well as lower proportion of PI-Annexin V double positive cells	1. plasma uric acid levels were increased at 2, 6 hours, and 1 day after radiation exposure 2. Blocking uric acid generation before and after 2 Gy radiation resulted in the decreased inflammasome activation	(44)
C57BL/6 female mice (8 weeks old)	75 Gy	Left lung	2 and 3 weeks	1. The expression of NLRP3 inflammasome-related genes (Nlrp3, Il1a, Il-1b, and Casp1) in lung tissue were increased 3 weeks after radiation		(46)
C57BL/6 female mice (6 weeks old)	75 Gy	Left lung	21 days	Increased mRNA levels of inflammasome related genes (Nlrp1, Nlrp3, Il-1b, and Casp1) in irradiated lung tissue		(49)
Male C57/6 mice	7.2 Gy, delivered in 5 days	total body irradiation	14 days	1. IR increased mRNA and protein levels of IL-1β and NLRP3 in thymus and spleen 2. Strong increases of IL-1β protein levels in intestine tissue as well as serum		(50)
C57BL/6 mice, NLRP3 macrophage-specific knockout mice	14 or 16 Gy	Whole-body radiation	7 days	1. 16 Gy radiation: all NLRP3-deficient mice died on day 6, 20% of WT mice survived until day 15 2. 14 Gy radiation: on day 15, 75% WT mice and 50% NLRP3-deficient mice survived	1. Higher level of ROS generation in the colon of NLRP3-deficient mice than in that of WT mice 2. NLRP3-deficient mice had a lower expression of barrier protein (ZO-1, E-cadherin, calaudin-2) than WT 3. cGAS-STING activation: elevated IFN-β levels in serum of NLRP3-deficient mice compared to WT. With increased p-TBK-1 and p-IRF3 protein levels in colon tissue	(51)
3-month-old C57BL/6 mice	40 Gy	The left thigh skin	8 weeks	1. mRNA and protein levels of NLRP3, caspase-1, and IL-1β were significantly increased in irradiated skin tissue	1. Increased serum 8-OHdG levels and skin γH2AX expression levels were detected 4 weeks after radiation 2. radiation causes increase in serum ROS levels as well as 4-HNE and 3-NT in skin tissue	(42)

ROS, reactive oxygen species; LPO, lipid peroxidation; GPx, glutathione peroxidase; GSSG, glutathione disulfide; GSH, glutathione; GR, glutathione Reductase; iNOS, inducible nitric oxide synthase; i-mtNOS, mitochondrial iNOS; BALF, bronchoalveolar lavage fluid; MDA, malondialdehyde; SOD, superoxide dismutase; Mn-SOD, manganese-dependent superoxide dismutase; PI, propidium iodide; p-TBK-1, phosphorylated TANK binding kinase 1; p-IRF3, phosphorylated interferon regulatory factor 3; 8-OhdG, 8-hydroxy-2’-deoxyguanosine; 4-HNE, 4-hydroxynonenal; 3-NT, 3-Nitrotyrosine.

**Table 2:** *In vitro* studies involving radiation-induced NLRP3 inflammasome activation.

Cell line	Radiation dose	Observation period	NLRP3 activation-wise results	Possible connections	Reference
THP-1 cells	2 Gy	1, 4, 6 hours	1. 2 Gy radiation increased protein expression levels of NLRP3, cleaved-caspase-1, IL-1β and IL-18 2. ROS production was increased in 1, 4 and 6 hours after radiation treatment	1. NAC (ROS inhibitor) treatment significantly decreased IL-18 and IL-1β protein levels in the supernatants of the THP-1 cells	(40)
primary BMDMs from C57BL/6J mice	5, 10, 20 Gy	3 or 24 hours	1. In 24 hours, 10 and 20 Gy radiation increased pyroptosis in a dose-dependent manner, NLRP3 knockout prevented radiation-induced pyroptosis 2. In 24 hours, 5 Gy radiation significantly induced the release of IL-1*β*, IL-18 and IFN-*γ*, 10 and 20 Gy radiation additionally increased the production of TNF-*α*, IL-1*α*, IL-12p40 and MCP-1 3. In 3 hours, 10 and 20 Gy radiation induced caspase-1 cleavage 4. In 3 hours, no significant changes were observed in mRNA levels of NLRP3 inflammasome-related genes (*Nlrp3, caspase-1* or *IL-1β*)		(48)
pulmonary microvascular endothelial cell, flow adapted (Mimicking *in vivo* vasculature)	①γ radiation: 0, 0.25, 0.5, 1 Gy ②low LET proton radiation: 1 Gy ③high LET proton radiation: 0.25, 0.5 Gy ④Mixed Field Gamma and Proton Radiation: 0.75 Gy	24 hours	1. 1 Gy low LET proton radiation significantly increased NLRP3 and ICAM-1 expression levels 2. 0.25 Gy or 0.5 Gy high LET proton radiation significantly increased NLRP3 and ICAM-1 expression levels 3. Mixed field gamma and proton radiation exposure induced robust increases in both NLRP3 and ICAM-1 expression	1. 0.25 and 0.5 Gy γ radiation causes mRNA levels of antioxidant gene, HO-1, NQO1, and GSTM1 2. IR significantly increases ICAM-1 expression in a dose-dependent manner 3. Mixed field radiation exposure induced extensive cell death	(52)
microvascular brain endothelial cells	2, 2.5, 5, 7.5, 10 Gy	24 hours	1. NLRP3 mRNA and protein expression level was induced 24h post IR, with no linear dose dependency 2. IR higher than 2 Gy causes pyroptosis with dose-dependency 3. IR higher than 2.5 Gy significantly increases the mRNA level of caspase-1 in 24 hours, as well as elevated the caspase-1/pro-caspase-1 ratio 4. IR higher than 5 Gy causes increase of ASC protein level, and 7.5 Gy for GSDMD protein level 5. IR higher than 5 Gy up-regulated mRNA and protein levels of IL-1β and IL-18 in 24 hours, up-regulation of cleaved-/pro-interleukin ratio in higher than 5 Gy for IL-18, and in higher than 2.5Gy for IL-1β		(53)
Human umbilical cord blood-derived mesenchymal stem cells	0, 2, 4, 8 Gy	24 hours	1. 4 Gy radiation increased NLRP3 mRNA and protein levels 2. radiation dose-dependently increased IL-1β mRNA and protein levels	1. the knockdown or inhibition of Sirt1 significantly enhanced radiation-induced IL-1β expression.	(54)

low/higher linear energy transfer (LET) protons (3–4 or 8–10 keV/µm, respectively); IR, ionizing radiation; THP-1, human monocytic leukemia cell line; BMDM, bone marrow-derived macrophages.

## Damage caused by radiation-induced inflammasome

The main manifestations of radiation injury are often two-fold. Firstly, in the acute stage, the irradiated tissues exhibit typical inflammatory responses. The clinical examples include radiation-induced pneumonitis (40), oral mucositis (41), and enteropathy (55, 56). The late stage of radiation injury is often related to chronic inflammation and fibrotic pathology, such as pulmonary fibrosis (40), osteoradionecrosis of the jaws (which is also considered a fibrotic lesion) (57, 58), and radiation-induced vasculopathy (30, 59, 60). The two-stage clinical manifestations suggest the existence of a dynamic and progressive pathology, in which NLRP3 inflammasome may have certain contributions.

### Excessive cell death and tissue dysfunction

Cell death is a common result of inflammasome (over) activation. Regulated-cell-death, like apoptosis and pyroptosis, was discovered to play a part in the pathogenesis and progression of radiation-induced injuries (34, 48). Pyroptosis is well known to lie downstream of inflammasome activation and is executed by GSDMD. Inflammasome-activated caspase-1 (also other inflammatory caspases like -4, -5, -11) cleaves GSDMD into N- and C-terminal fragments. GSDMD-N then translocate to inner leaflet of membrane and bind to phospholipids, oligomerize in membranes to form pores, allowing the release of cell contents like mature IL-1β, IL-18, TNF-α and HMGB1, followed by pyroptotic cell death. GSDMD pores may further enhance NLRP3 inflammasome activation by promoting K^+^ efflux, forming a positive-feedback (61–63). Moreover, with research in cell death continuing to abound, it has been recognized that apoptosis may also be induced by inflammasomes. On the one hand, in cells with low expression of GSDMD, caspase-1 initiates apoptosis either through activating Bid (an extrinsic apoptosis pathway mediator) or through processing caspase-7, rather than inducing pyroptosis (28). On the other hand, ASC specks may also recruit and activate caspase-8 and undergo apoptosis (64). It has also recently been demonstrated that apoptosis may enhance NLRP3 inflammasome activation by cleaving pannexin-1 to induce K^+^ efflux (65). To recapitulate briefly, both pyroptosis and apoptosis lie downstream of inflammasome activation, depending on the expression of GSDMD, and both may contribute to further inflammasome assembling (28).

Cell death caused by inflammasome activation may present diverse results among various types of tissues. In macrophages, for example, radiation acutely activates NLRP3 inflammasome signaling both *in vivo* and *in vitro*. Low dose (<5 Gy) radiation stimulates macrophages to secrete proinflammatory molecules while high dose (>10 Gy) promotes its pyroptosis (48), which may justify immune cell loss and myeloid suppression after extreme radiation exposure or high-dose RT (66–68). Moreover, the epithelial tissues, such as oral mucosa, gut mucosa, as well as lung epithelium, are particularly susceptible to ionizing radiation. Inflammasome activation and pyroptosis have been observed in these tissues after exposure to radiation (47, 48, 53, 69). Excessive cell death in epithelium may lead to the breakdown of barriers such as skin, gut mucosal barrier, and alveolar epithelial barrier. Compromised barriers allow pathogenic microbes to invade inflammatory tissue, exacerbating immune response and tissue damage (17, 70). LPS from Gram-negative bacteria may also contribute to the inflammasome cascade through non-canonical NLRP3 inflammasome activation, thereby leading to even deteriorated conditions (29). Equally important, immoderate cell death in vascular endothelial cells causes vascular dysfunction, leading to increased permeability, impaired vascular tone, and altered blood homeostasis, aggravating already severe damage caused by radiation (10, 53).

In the background of radiation injury, cell death should be contained within a reasonable range to minimize normal tissue injury, since excessive cell death leads to tissue dysfunction of various kinds, adding to more unwanted events. Targeting solely against apoptosis or pyroptosis, however, may not fulfill the designated goal, since the two cell death machineries compete to be performed (64). Given that both pyroptosis and apoptosis lie downstream of inflammasome activation, it is therefore preferable to target this upstream pro-inflammatory signaling. In other words, early preventive measures are necessary to prevent irreversible damage to cells, which will be discussed in the later section.

### Onset of chronic radiation injury

Inflammasome activation enables the maturation and release of IL-1β and IL-18, thus functioning as an immune regulator in radiation-induced injury. Both IL-1β and IL-18 are multi-functional immune modulator and inflammatory amplifier, mediating the initiation of innate and adaptive immune response (17, 71). Cascaded inflammation may lead to excessive production of numerous proinflammatory cytokines (like IL-1, IL-6, TNF-α, etc) and chemokines, together with protracted radiation stimulation, may give rise to the establishment of chronic inflammation and tissue injury (14, 72).

It is widely accepted that the chronic phase of radiation-induced tissue injury involves damage repair and tissue remodeling. However, when accompanied with persistent oxidative stress and non-resolving inflammation, damaged tissues may not heal properly and result in fibrotic lesions (14, 73). Radiation-induced fibrosis (RIF) is characterized by increased collagen deposition, poor vascularity, and scarring (57). The current understanding on the development of RIF entails the augment of TGF-β signaling, vascular injury and hypoxia, chronic inflammation, and finally, the activation of myofibroblasts that mediates aberrant tissue remodeling (74).

The contributions of NLRP3 inflammasome to various types of fibrosis are gradually recognized (75). Mainly, IL-1β and IL-18 possess the capability to directly induce collagen synthesis in fibroblasts, or *via* interacting with SMAD signaling and promote epithelial-mesenchymal-transition, as well as inducing TGF-β through activating NF-κB (75, 76). NLRP3 is also demonstrated to directly participate in fibrosis by augmenting TGF-β signaling, independently of its inflammasome property. Though most studies on the pro-fibrosis property of inflammasome were conducted on the model of chronic kidney disease or liver fibrosis (77, 78), we may well extrapolate the potential contribution of inflammasome to RIF based on these understandings.

TGF-β, a prominent pro-fibrosis mediator, which activates fibroblasts through the SMAD signaling, is in the spotlight of studies on RIF (79, 80). The production and the function of TGF-β relate to inflammasome activation in several respects. As is discussed afore, ROS is acutely and persistently produced upon irradiation, which not only activates NLRP3 inflammasome, but is also discovered to promote the production and enhance the signaling of TGF-β (81). Moreover, the inflammasome-mediated vascular injury and dysfunction may help establish a hypoxic environment, which further augments the production of free radicals. A hypoxic state also leads to increased HIF-1a signaling and promotes various pro-fibrotic mediators (74). Potentiated local inflammation as well contributes to fibrogenic processes (14).

Further research is recommended to unravel the exact role of NLRP3 inflammasome in RIF, as regulatory strategies of the complex are widely studied and practiced. In case there is a strong connection between NLRP3 inflammasome and RIF, the latter may be prevented by targeting the former.

Although we have stressed on the detrimental aspects of inflammasome-induced response, the beneficial aspects of inflammation should not be neglected. For instance, inflammasome-induced cell death may assist the clearance of severely damaged cells. Moreover, the attracted immune cells also serve to protect tissues from barrier breakdown and potential infections, and scavenge dead cells and initiate tissue repair and remodeling. They only have a detrimental effect if the balance of inflammation is out of control. Hence, in-depth characterization of these machineries and designing of strategies specifically against undesired injuries is of vital importance.

## Targeting inflammasome in radiation injury

As previously explained and analyzed, NLRP3 inflammasome plays a key role in radiation injury, as it is activated by radiation, and then mediates at least a certain range of injuries. In other words, radiation is the one that gives the order, it is the inflammasome that responds and kills—functioning as the executioner. Fortunately, there exist ample options to target NLRP3 inflammasome and restrain it from causing tissue injuries.

Radiotherapy dose is directly related to the achievement of desired local-regional control of cancer. However, severe adverse effects of radiation are the main reason for reducing the radiotherapy dose, which is especially the case when the tolerance of “organ at risk” is taken into account (9, 82). For example, if the unwanted effects (indicative symptoms or pathological changes for endpoints) of organ at risk arrive too early, the designated RT dose may not be accomplished, leading to increased risk of recurrence. Moreover, even in the absence of acute symptoms, radiologists need to be cautious when considering the late adverse effects of organs that are sensitive to radiation, restraining the efficacy of radiotherapy. Hence, strategies for counteracting the adverse effects of radiation should be explored in order to achieve a higher upper limit of radiotherapy dose.

We here propose the necessity of early intervention against inflammasome in radiation injury. The definitions of the adjective “early” are two-fold, comprising both macroscopic and microscopic perspectives. Firstly, by referring to early intervention at the clinical level, we intend to emphasize the active prevention of radiation injury, rather than passively dealing with end-stage problems. Studies focused on other NLRP3-associated clinical problems, such as post-myocardial infarction fibrosis and autoimmune diseases, have discovered that inhibition of NLRP3 inflammasome in the early phases of diseases may reduce the occurrence of severe, late-stage lesions (83, 84). Moreover, at the molecular level, the activation and downstream effects of NLRP3 inflammasome are sequential, consecutive processes, which suggests that if the inhibition is designated at the upstream of the pathway, such as transcriptional (priming) repression, sensor protein inhibition, and removal of stimulants, the entire signalling cascade may be suppressed. For example, inhibiting the activation of inflammasome signalling was shown to reduce macrophage pyroptosis (48), as well as preserve the integrity of the epithelial barrier (50, 73), thereby reducing the occurrence of tissue dysfunction.

### Conventional regulation strategies and early intervention

Conventional targeting against NLRP3 inflammasome include stimulant removal, transcriptional regulation (41, 49, 50, 54), activation inhibition (84, 85), effector protein (namely caspase-1) targeting (86), and product targeting (mostly against IL-1β and GSDMD) (30, 87, 88). The representative therapeutics based on these concepts are briefly summarized in **Table 3**.

**Table 3:** Conventional therapeutic strategies for regulating the NLRP3 inflammasome.

Medication	Application in disease therapy	Mechanism	Stage	Reference
anakinra	CAPS and rheumatoid arthritis	IL-1R antagonist	Clinical trial	(86)
Canakinumab	CAPS, atherosclerotic diseases, arthritis and gout	IL-1β-neutralizing antibody		(86)
rilonacept	CAPS	decoy receptor that binds both IL-1β and IL-1α		(89)
Tranilast	allergy, asthma and hypertrophic scars	binds the NACHT domain of NLRP3, affects the oligomerization (without affecting the ATPase activity)		(20)
VX-740 and VX-765	murine osteoarthritis, delayed-type hypersensitivity	reversible caspase-1 inhibitor	Pre-clinical	(20, 86)
Glyburide	efficiently prevent endotoxic-shock-induced lethality	mechanism unknown, though functions downstream of the P2X7 receptor and upstream of NLRP3		(90)
MCC950	CAPS and EAE	blocks ASC oligomerization; directly binds to the NACHT domain and changes NLRP3 conformation		(86, 90–92)
BHB	Muckle–Wells syndrome, familial cold autoinflammatory syndrome and urate crystal–induced peritonitis	preventing potassium efflux and reducing ASC oligomerization and speck formation		(90, 93)
JC171	delayed the progression and reduced the severity of multiple sclerosis	interfering interaction with ASC		(86)
CY-09	CAPS and type 2 diabetes	directly binds to NLRP3 NACHT domain and inhibits NLRP3 ATPase activity		(83)
OLT1177	degenerative arthritis	directly binds to NLRP3 and inhibits ATPase activity		(94)
ibrutinib	ischemic brain injury, metabolic inflammation and SCD	a BTK inhibitor, suppresses NLRP3 activation and IL-1β release		(95–97)
Disulfiram	LPS-induced sepsis	blocking GSDMD pore formation		(98)

CAPS, cryopyrin-associated periodic syndromes; VX-740, Pralnacasan; VX-765, Belnacasan; MCC950, CP-456773; EAE, experimental autoimmune encephalitis; BHB, β-hydroxybutyrate; SCD, sickle cell disease; BTK, Bruton tyrosine kinase.

**Figure 2:**
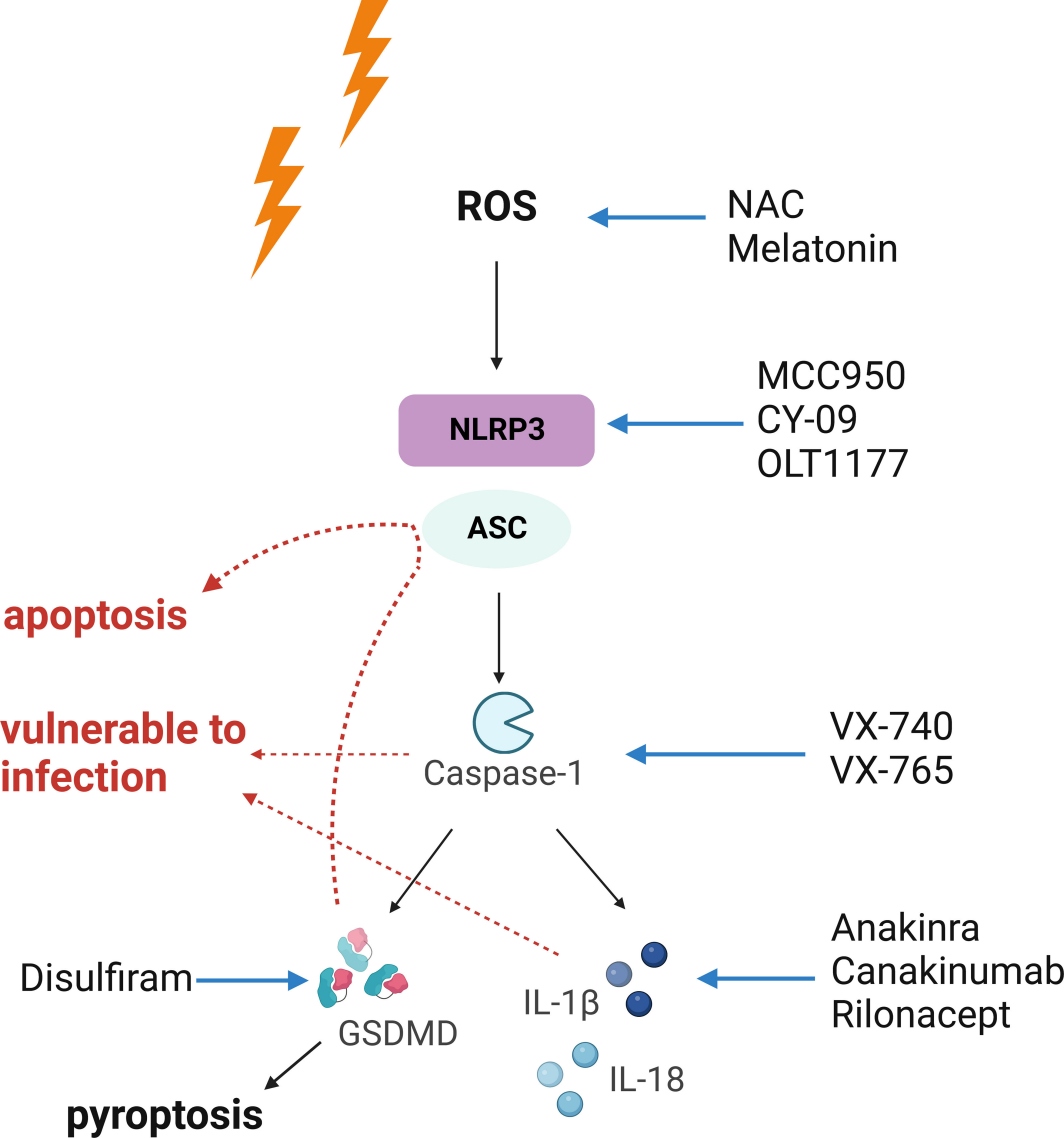
The necessity of early intervention against radiation-induced injury. The NLRP3 inflammasome acts as an executioner in radiation-induced tissue injury. Upon activation, it triggers a cascaded reaction, which indicates that interventions targeting upstream of the signaling may yield the most desirable outcomes. In comparison, the results of downstream intervention may be accompanied with unwanted effects. Red arrows indicate unwanted effects of strategies suppressing pyroptosis, caspase-1, or IL-1β. Blockade of pyroptosis may lead to activation of apoptosis or other death pathways. Also, caspase-1 is required for the proper function of other inflammasomes, and blockade at this point may shut down the entire inflammasome signaling, leaving the organism vulnerable to infection. Suppressing IL-1β also increases the risk of infection. Both **Figures 1**, **2** were created with BioRender.com.

Early intervention against NLRP3 inflammasome may lead to better outcomes. When solely considering the manipulation of NLRP3 inflammasome, it is noteworthy that these strategies may yield various results, as they are designed for different stages of the signaling cascade. First and foremost, elimination of ROS with antioxidants leads to suppression of the entire pathway accompanied by reduction in free radical-induced oxidative damage, which is the most desired situation (8, 52, 99). Moreover, modifying the NLRP3 inflammasome *via* transcriptional regulation or direct inhibition of activation (suppressing NLRP3 protein function) may generate similar outcomes, as they both prevent the oligomerization of the inflammasome complex. However, the rest of the mentioned strategies bear extra unwanted effects, since different types of inflammasomes share an identical set of downstream signaling. For example, inhibition of caspase-1 may interrupt the proper functioning of other inflammasomes (such as AIM2 and NLRC4 inflammasomes) as well, increasing the susceptibility to infection. Specific targeting of IL-1β may lead to increased incidence of infection too, as observed in the CANTOS trial (86, 100). Playing downstream of inflammasome activation, pyroptosis may be precluded *via* disrupting the upstream signaling. However, manipulation of the effector for pyroptosis, GSDMD, may not redeem the dying cells, as other programmed cell death pathways may substitute pyroptosis leading to cell death (64). To summarize these machineries, it is obvious that the more upstream the interventions are aimed at, the greater the outcomes may yield. Our proposition on early intervention against NLRP3 inflammasome is illustrated in **Figure 2**.

### Transcriptional and translational regulation

NF-κB is a multi-functional regulator that controls diverse cellular processes like immune response, proliferation and cell survival, etc. Various PAMPs, DAMPs, and some endogenous molecules may give rise to NF-κB activation through triggering TLRs, TNFR1 and IL-1R signaling (101). In respect of radiation-induced response, NF-κB upregulates diverse pro-survival (such as antioxidant enzymes, anti-apoptotic proteins and growth factors) and pro-inflammatory (various cytokines, chemokines and adhesion molecules) genes expression (102), thus exhibiting either protective and damaging effects.

NF-κB contributes to activation of NLRP3 inflammasome by providing NLRP3 components and pro-IL-1β. Targeting NF-κB signaling may disrupt priming and ameliorate NLRP3 inflammasome activation. Targeting IRAK4, a mediator of TLR-NF-κB signaling, may partially suppress NLRP3 transcription (24). Resveratrol, a natural non-flavonoid polyphenolic as well as an antioxidant, is shown to mitigate radiation-induced NF-κB activation through regulating SIRT1 (50, 54). Similarly, Rosiglitazone, an agonist of PPARγ, is able to suppress the expression and release of NLRP3, caspase-1, and, IL-1β through attenuating NF-κB signaling (35, 103).

Post-transcriptional regulation of NLRP3 is mainly mediated by a group of miRNA and long non-coding RNA (lncRNA). miR-133b, miR-20b, miR-223 and others mediate the silencing of the NLRP3 gene. lncRNAs interact with miRNAs and can either promote or attenuate inflammasome signaling depending on tissues and pathologies (23, 83). Moreover, NLRP3 inflammasome has also been targeted post-translationally through regulating its phosphorylation, ubiquitination, and their reverse processes. For instance, targeting NLRP3 phosphorylase like JNK1 and promoting NLRP3 degradation *via* deubiquitylation inhibitor (23, 86) has been applied in inflammatory diseases, and is also a potential therapeutic strategy in radiation injury.

### Modulation of NLRP3 inflammasome through intersectional pathways

NLRP3 inflammasome has a complex regulatory mechanism, which may interact with key proteins involved in other aspects of cellular physiology. It is therefore possible to regulate NLRP3 inflammasome through modulating these intersecting pathways.

The competition of DDX3X between NLRP3 inflammasome and stress granules (SGs) provides an option to regulate NLRP3 inflammasome through modulating the formation of the latter. DDX3X is a necessary component of the NLRP3 inflammasome, which is also involved in the initiation of SGs assembly. After formation of SGs, DDX3X is sequestered within the complex, leaving NLRP3 inflammasome inactivated (104–106). Such interaction is promising for designing therapeutic strategies. For instance, pharmacological induction of SGs was shown to protect cochlea cells against ototoxic drugs challenge (107), and a similar strategy may be explored and applied in radioprotection as well.

Autophagy is also known to interact with NLRP3 inflammasome, and therefore, may serve as a potential regulatory strategy for radiation-induced inflammasome as well. Autophagy plays a particularly important role in the recycling and removal of damaged cell components (108). In the context of radiation injury, the clearance of damaged mitochondria through autophagy or mitophagy (a form of autophagy for selective removal of dysfunctional or redundant mitochondria) may avoid NLRP3 inflammasome activation (83, 108–110). Correspondingly, in a recent study, induction of autophagy was found to ameliorate radiation-induced enteropathy by promoting phagocytosis of the NLRP3 inflammasome, which further confirmed our hypothesis (111).

### Novel therapeutics

Dysbiosis of resident microbiota may contribute to enhanced immune response (112, 113). Moreover, barrier breakdown in radiation injury may allow the translocation of pathogenic microbes and increased infiltration of immune cells, which further exacerbates tissue damage (39, 114–116). Inspired by these observations, researchers have utilized commensal microbiota against radiation injury and obtained fruitful outcomes. Microbiota transplantation has been shown to alleviate radiation injury of the intestinal and oral mucosa; the results also indicated that transplanted microbiota can regulate immune response and further influence the outcomes of irradiated tissue (117, 118). Though the exact relationship between radiation injury and NLRP3 inflammasome has not yet been illustrated, fecal microbiota transplantation was found to inhibit the expression of inflammasomes components (NLRP3, ASC, caspase-1, and IL-1β) in rat brain, thus ameliorating stress-induced depression-like behaviors (119, 120). The effectiveness of fecal microbiota transplantation in alleviating radiation injury and suppressing NLRP3 inflammasome points towards a connection between commensal microbiota and radiation-induced inflammasome. Efforts should be made to better comprehend their interactions and design pertinent therapeutic strategies.

Mesenchymal stem cell (MSC) therapy has emerged as a promising therapeutic modality for multiple diseases considering their convenient isolation and culture, low immunogenicity, regenerative and multiple differentiation abilities, and potent immunomodulatory capacities. MSC therapy has been shown to attenuate radiation-induced brain damage by suppressing microglia pyroptosis, reducing ROS production, and NLRP3 inflammasome activation (121). Also, modified MSCs serve as a practical assistant in gene therapy for radiation injury, which efficiently deliver target genes to the injured sites and alleviate radiation injury (122). However, MSCs may transform into malignant cells; hence, it is necessary to evaluate potential side effects and to prevent them in advance.

Exosomes have been applied to the treatment of radiation injury as vectors for therapeutic agents (123). Moreover, recent evidence also suggests that the secretions of exosomes interact with inflammasome pathology (124, 125), which may be involved in MSCs-mediated therapies for radiation injury (126). As a therapeutic carrier, exosomes are considered safer than cell therapy because of their lower immunogenicity. Moreover, exosomes have almost no cytotoxicity, better storage stability, antiserum aggregation ability, and biological activity (123). Based on the above-mentioned advantages, exosomes are potential promising carriers for delivery of various therapeutic loads, such as siRNA and miRNAs against inflammasome components, to the desired target sites.

## Conclusion and future perspectives

As discussed in this review, NLRP3 inflammasome is one of the executioners of radiation-induced tissue injury, which mediates a range of common radiation-induced illnesses. With ongoing advances in the research on radiation-induced inflammasome, the prospects of targeting inflammasome as a preventive measure against radiation injury appear practicable. However, there are some unsolved questions in this field. For example, the concepts this review proposes mainly focuses on the detrimental aspects of the inflammasome in radiation injury; however, the inflammasome is also responsible for tumor immunity, pathogen clearance, and initiating tissue repair in some cases (100). More importantly, due to the diverse expression patterns of the NLRP3 inflammasome, its exact roles among different forms of injuries may vary. Proper targeting of inflammasomes should be elaborate and precise, which is why there is a need for further in-depth research.

Radiotherapy is an effective treatment modality for various malignancies in clinical practice. Nonetheless, the side effects of ionizing radiation restrict its use to some extent. This is particularly the case when high doses are required for the treatment of advanced, unresectable malignancies. More importantly, the currently used measures for protecting normal tissue are generally passive and are typically implemented after the development of injuries. In between prevention, mitigation, and treatment, there is no doubt that the first one stands out as the most ideal strategy (127).

However, the reality of radioprotectants is frustrating. According to the 2008 ASCO guidelines for the clinical use of radiotherapy protectants, only dexrazoxane, doxorubicin, amifostine, and palifermin are recommended under several specific circumstances (128). This is because of the inherent challenges in the development of an effective and harmless radioprotectant. Till date, no clinical trials have been conducted in the context of radiation-induced inflammation, suggesting the inadequacy of research in this field.

Based on the analysis of the mechanism and function of the NLRP3 inflammasome in radiation injury, this review may provide insights for developing better therapeutic strategies against radiation-induced injury to normal tissues, as well as highlight the critical role of this machinery. In-depth characterization of the physiology of radiation injury and its counteracting response is crucial for the further advancement of radiotherapy.

## Author contributions

HC, LC, MH, and JH contributed to design, drafted, and critically revised the manuscript. ZC and XY contributed to conception, design, and critically revised the manuscript. All authors contributed to the article and approved the submitted version.

## Funding

This work was supported by the National Nature Science Foundation of China (No. 82173458) and College Students’ Innovative Entrepreneurial Training Plan Program (No. X202012121296 & No. X202012121293).

## Acknowledgments

The illustrations this review presented were created with BioRender.com.

## Conflict of interest

The authors declare that the research was conducted in the absence of any commercial or financial relationships that could be construed as a potential conflict of interest.

## Publisher’s note

All claims expressed in this article are solely those of the authors and do not necessarily represent those of their affiliated organizations, or those of the publisher, the editors and the reviewers. Any product that may be evaluated in this article, or claim that may be made by its manufacturer, is not guaranteed or endorsed by the publisher.

## Glossary

**Table:**

DSBs	double-strand DNA breaks
ROS	reactive oxygen species
RNS	reactive nitrogen species
DDR	DNA-damage response
MRN	Mre11-Rad50-Nbs1
ATM	ataxia telangiectasia mutated
H2AX	histone variant 2AX
NF-κB	nuclear factor kappa-B
IL-1β	interleukin-1β
IL-18	interleukin-18
NLRP3	nucleotide-binding oligomerization domain (NOD)-like receptors, pyrin domain-containing protein 3
AIM2	absent in melanoma 2
PAMPs	pathogen-associated molecular patterns
DAMPs	damage-associated molecular patterns
ASC	apoptosis-associated speck-like protein containing a CARD
caspase	cysteinyl aspartate specific proteinase
GSDMD	gasdermin-D
LPS	lipopolysaccharide
ETC	electron transfer chain
TXNIP	thioredoxin-interacting protein
Trx-1	thioredoxin-1
NAC	N-acetylcysteine
mtDNA	mitochondrial DNA
RIF	Radiation-induced fibrosis
SGs	stress granules
MSC	mesenchymal stem cell
